# Nails in older adults

**DOI:** 10.1080/07853890.2024.2336989

**Published:** 2024-05-13

**Authors:** Samantha Jo Albucker, Jade Conway, Shari R. Lipner

**Affiliations:** a Department of Dermatology, Tulane University School of Medicine, New Orleans, LA, USA; b Department of Dermatology, NY Medical College, Valhalla, NY, USA; c Department of Dermatology, Weill Cornell Medicine, New York, NY, USA

**Keywords:** Nail disorders, older adults, age, senile, geriatric nail conditions

## Abstract

As the world’s population of adults greater than 60 years old continues to increase, it is important to manage nail disorders that may impact their daily lives. Nail disorders may have significant impact on quality of life due to decreased functionality, extreme pain, or social embarrassment. In this review, we discuss nail disorders affecting older patients, including physiologic, traumatic, drug-induced, infectious, environmental, inflammatory, and neoplastic conditions. Diagnosis of these conditions involves a detailed history, physical examination of all 20 nails, and depending on the condition, a nail clipping or biopsy and/or diagnostic imaging. Nails grow even more slowly in older adults compared to younger individuals, and therefore it is important for accurate diagnosis, and avoidance of inappropriate management and delay of treatment. Increased awareness of nail pathologies may help recognition and management of nail conditions in older adults.

## KEY MESSAGES

- Nail disorders are common amongst older adults and may cause decreased functionality, pain, psychosocial problems and impact quality of life.
- Many nail conditions, both physiologic or pathologic, may have similar presentation in older adults. Confirmation testing is important to avoid inappropriate or delayed treatment.
- The increased frequency of comorbidities, drug interactions, polypharmacy, and mental or physical limitations with aging must be considered when managing care of older patients with nail disorders.

## Introduction

The population of adults ages 60 years old is estimated to double to 2.1 billion, and 80 years and older is expected to triple to 426 million by 2050 [1], highlighting the need to diagnose and treat nail disorders that affect the daily lives of older adults in terms of functionality, pain, or social embarrassment. Moreover, more generally dermatologists serve an important role in helping patient navigate the process of healthy aging as they address factors that fundamentally affect both physiologic and pathological processes faced by older adults [2]. In this review, we aim to discuss a breadth of nail disorders affecting older patients Table 1, including physiologic, traumatic, drug-induced, infectious, environmental, inflammatory, and neoplastic nail changes.

**Table 1.** Summary of common nail changes in older adults.

Diagnosis	Disease characteristics
Onychogryphosis	Thickening, hypertrophy, and brown opaque nail plate discoloration, most frequently affecting the great toenails [4,5,11]. Presents as ‘ram’s horn-like’ or ‘oyster-like’ with transverse striations, often associated with trauma, nail surgery, foot-to-shoe incompatibility, or hallux valgus [3,5,11].
Onychocryptosis	Ingrown toenail presenting with pain at rest, ambulation, or with pressure [5,8]. May be caused by trauma, weight fluctuation, hyperhidrosis, poor nail cutting, onychotillomania, history of nail surgery, obesity, bony abnormalities, onychomycosis, foot-to-shoe incompatibility, or hallux valgus [5,8].
Onychauxis/Pachyonychia	Localized nail plate hypertrophy with hyperkeratosis, discoloration, and decreased translucency with or without subungual hyperkeratosis and debris [3,5,6].
Onychophosis	Hyperkeratosis of the lateral or proximal nail folds, between the nail fold and nail plate, or subungual area [3,5]. The great and fifth toenails are most commonly affected, likely because they are most often subject to trauma [3,5].
Onychoclavus	Subungual heloma or corn presenting as hyperkeratosis with or without melanonychia overlying the nail bed typically affecting the distal great toenail [3,5]. May be due to trauma, foot-to-shoe incompatibility, digits flexi, hallux valgus, hammer toe deformity, or rotated fifth toes [5,6].
Subungual Hematoma and Splinter Hemorrhages	Violaceous, black nail plate discoloration that migrates distally with nail growth most likely due to trauma [5,17]. May be accompanied by onycholysis [5,17]. Splinter hemorrhages may be a sign of nail psoriasis [5].
1. Beau’s lines 2. Onychomadesis 3. Retronychia	1. Transverse grooves in the nail plate caused by a temporary decrease of mitotic activity of nail matrix keratinocytes [20,21]. 2. The complete nail plate separation and shedding with slow longitudinal growth rate after a traumatic event [20]. 3. The malalignment of the nail plate resulting in growth of the nail plate proximally towards the nail fold [24].
Onychomycosis	Fungal infection of the nail unit causing yellow nail plate discoloration, thickening, onycholysis, crumbling, and subungual hyperkeratosis [30].
Periungual and Subungual Warts	Caused by HPV [44]. Immunosuppression is a major risk factor [5].
Acute Paronychia	Bacterial infection of the nail folds, most commonly caused by *Staphylococcus aureus* [3,5]*.* Presents with erythema, tenderness, and localized pus formation [3,5]. Most likely due to trauma and typically affects one nail [3,5].
Chronic Paronychia	Nail fold inflammation with erythematous and swollen nail folds with cuticle loss [3,5].
Brittle Nail Syndrome (BNS)	Increased nail plate fragility, most frequently seen in women [48,48]. Presents with onychoschizia, onychorrhexis, or splitting [4,6].
Nail Psoriasis	Nail matrix psoriasis presents with pitting, crumbling, leukonychia, and red spots in the lunula [52]. Nail bed psoriasis presents with splinter hemorrhages, onycholysis, oil drops and nail bed hyperkeratosis [52].
Nail Unit Melanoma	Longitudinal melanonychia, or longitudinally oriented brown to black band that extends the length of the nail plate [61]. May present as amelanotic [63–65]. Concerning findings are bands measuring > 3 mm in width, band widening, heterogeneity in color, bleeding, and nail splitting [62]. Hutchinson’s sign is periungual pigment involving the nail folds or hyponychium is often concerning for invasive melanoma [63].
Bowen’s Disease	An ulcerated, hyperkeratotic lesion often accompanied by erythema, scaling, and crusting [69]. Risk factors include HPV, trauma, ionizing radiation, smoking, arsenic exposure, and chronic paronychia [6,71].
Myxoid Cyst	Skin-colored to translucent, smooth, dome-shaped, and fluctuant nodules located distal to the interphalangeal joint [34,72]. Most commonly on the first three fingers [34,72].

## Physiologic changes

Physiologic nail changes in older adults include alterations in color, thickness, contour, texture, growth rate, and chemical composition, which may be due to decreased circulation and changes in elastic or connective tissue [3,4].

Nails of older adults often appear dull, opaque, or pale with white (leukonychia), yellow, brown, or gray discoloration [5]. One type of physiologic leukonychia is Neapolitan nails, which resemble Neapolitan ice cream with a proximal white band and absent lunula, central pink band, and distal opaque band [3,5–7]. The bands remain stable with longitudinal nail growth. In a study of 258 patients >70 years old, 19% of patients had Neapolitan nails, which were associated with osteoporosis and thin skin (*p* < 0.05) [7].

Nail thickness is variable in older adults, with some presenting with an increase, decrease, or no change in nail thickness. While nails are normally smooth, texture changes associated with aging include increased longitudinal striations that are either superficial (onychorrhexis) or deep (ridging). Onychorrhexis is due to decreased nail matrix cell turnover rate. Other texture changes include transverse grooves, pitting, or trachyonychia (sand paper nails) [3].

**Figure 1.**
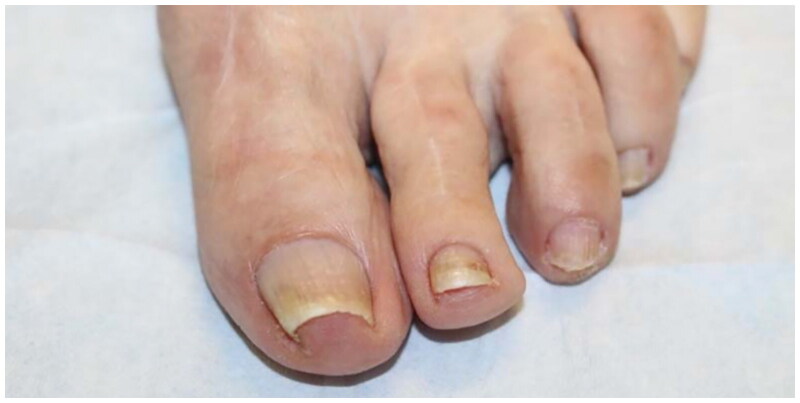
Pincer nail of the left first toenail in an 80-year-old woman. The lateral aspect of the nail plate is penetrating the periungual dermis of the lateral nail fold [8].

The nail contour of older patients has decreased longitudinal convexity with increased transverse curvature [3–6]. Other changes, though not necessarily physiologic changes, include koilonychia (spooning), nail plate flattening, and pincer nails (Figure 1) [3].

On average, toenails grow 1.0 mm/month and fingernails 3.0 mm/month. In a study of linear nail growth rate in 192 females and 79 males from 10–100 years old, nail growth decreased by 0.5% per year from 25–100 years old. The thumbnail decreased by 38% between the third and ninth decades. On average, males have faster growth rate until the sixth decade of life, but by the eighth decade females have faster growth rate [9].

Alterations to the chemical composition in nails of older adults include increased calcium and decreased iron [3]. Moreover, there is an increase in collagen cross-linking with aging, which may affect nail flexibility [10]. On histopathology, keratinocytes are larger and there is a greater number of keratinocyte nuclei remnants (pertinax bodies) [3,5]. The nail bed dermis demonstrates blood vessel thickening and degeneration of elastic tissue [3].

## Traumatic changes

### Onychogryphosis

**Figure 2.**
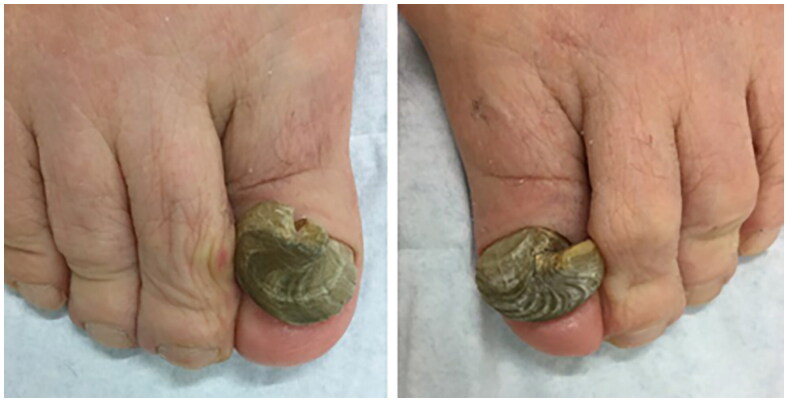
A 75-year-old female presented with painful bilateral great toenails for 10 years. Her nails grew slowly and were extremely difficult to clip. A full nail examination was significant for opaque yellow-brown thickening, hyperkeratosis, elongation, and increased curvature of the great toenails. Onychogryphosis can be differentiated from retronychia and onychomycosis by its spiral striated appearance [13].

Onychogryphosis is defined as thickening, hypertrophy, and brown opaque nail plate discoloration, most frequently affecting the great toenails [4,5,11]. It is a frequent problem in older patients, especially in those that are unable to maintain regular nail care. In a cross-sectional observational study of 173 patients (mean age at long term health facility: 85.0 ± 9.7 years, at special nursing home 1: 86.8 ± 7.2 years, and at special nursing home 2: 87.5 ± 7.1 years), prevalence of onychogryphosis was 17.9% [12]. The nail presents as ‘ram’s horn-like’ or ‘oyster-like’ with transverse striations, often associated with trauma, nail surgery, foot-to-shoe incompatibility, or hallux valgus [3,5,11]. Often the nails grow upward and laterally and the direction of growth can be directed by shoe pressure [3,5]. It is frequently associated with poor peripheral circulation (i.e. varicose veins, stasis dermatitis, and lower leg ulcers) [11]. Onychogryphosis can be distinguished from retronychia and onychomycosis by its spiral striations [13] (Figure 2). Prevention can be achieved with regular nail trimming and wearing comfortable shoes to relieve pressure and limit microtrauma. However, many older adults may be unable to maintain regular nail care, as they may have difficulty trimming their toenails due to mobility limitations [14]. Those who do not have access to help may develop the ‘long toenail sign,’ a potential indicator of difficulties with self-care [14]. Management includes electric filing and drilling for mechanical debridement, chemical nail avulsion *via* 40% urea or 50% potassium iodide under occlusion, or surgical avulsion with or without matriectomy [11]. After onychogryphosis treatment, patients may see normal nail growth or possible recurrences. Hence treatment may need to be repeated and nails should be kept short to prevent recurrence.

### Onychocryptosis

Onychocryptosis, or ingrown toenail, presents with pain at rest, ambulation, or with pressure [5]. It has a bimodal presentation, presenting between the first and third decades and then in older adults [8]. It may be caused by trauma, weight fluctuation, hyperhidrosis, poor nail cutting, onychotillomania, history of nail surgery, obesity, bony abnormalities, onychomycosis, foot-to-shoe incompatibility, or hallux valgus [5,8]. With trauma, constricting footwear, or expanding feet secondary to edema or weight gain, a nail barb or spicule can penetrate the nail fold as the nail plate grows [8]. For older patients with comorbidities that result in decreased sensation of feet/toes (i.e. diabetes mellitus, peripheral vascular disease, or arteriosclerosis), patients experience minimal pain and may present with infection, osteomyelitis, or gangrene [5,8]. Prevention can be achieved with regular nail trimming such that the nail plate is cut straight and the corners are beyond the distal edge of the lateral nail folds [8].

Treatment includes conservative approaches, such as taping, cotton packing, dental flossing, nail bracing (orthonyx technique), and super-elastic wiring. If conservative approaches fail, surgical approaches include partial/complete nail avulsion with or without matricectomy [5,8,15]. A systematic review of 18 studies that discussed patient-reported outcomes of onychocryptosis treatments demonstrated that patients receiving both nonsurgical and surgical interventions reported relatively high levels of patient satisfaction [16].

### Onychauxis

Onychauxis, or pachyonychia, is defined as localized nail plate hypertrophy with hyperkeratosis, discoloration, and decreased translucency with or without subungual hyperkeratosis and debris [3,5,6]. It may be due to overlapping/underlapping toes, foot-to-shoe incompatibility, digiti flexi, hallux rigidus, or hallux valgus [5], and may result in onycholysis, pain, and increased risk of onychomycosis [3]. Since onychauxis is sometimes misdiagnosed as onychomycosis and inappropriately treated with antifungals [5], confirmatory testing should be performed. Prevention can be achieved with regular nail trimming, while management includes electric filing, chemical nail avulsion *via* 40% urea, or surgical avulsion with or without matricectomy [3,5].

### Onychophosis

Onychophosis is defined hyperkeratosis of the lateral or proximal nail folds, between the nail fold and nail plate, or subungual area. It is common in older patients and the great and fifth toenails are most commonly affected, likely because they are most often subject to trauma. Risk factors include foot-to-shoe incompatibility, digiti flexi, hallux valgus, and rotated fifth toes. Preventative measures include wearing comfortable shoes and relieving pressure. Treatment includes nail debridement or application of keratolytics (i.e. urea 20%, ammonium lactate 12% or salicylic acid 6–20%) [3,5].

### Onychoclavus

Onychoclavus, a subungual heloma or corn, presents as hyperkeratosis with or without melanonychia overlying the nail bed typically affecting the distal great toenail [3,5]. It may be resemble benign melanocytic activation or malignant melanoma [4]. Onychoclavus may be due to trauma, foot-to-shoe incompatibility, digits flexi, hallux valgus, hammer toe deformity, or rotated fifth toes [5,6]. Since it is associated with subungual exostosis or chondroma, radiologic examination may be used to rule out an underlying bony abnormality [5]. Management includes avoiding tight-fitting shoes and wearing protective pads to relieve pressure, removal of hyperkeratotic tissue, and surgical correction of any osseous anomaly [3,5,6].

### Subungual hematoma and Splinter hemorrhages

Subungual hematomas are common in older patients and initially present as violaceous-black nail plate discoloration that migrates distally with nail growth. Sometimes, onycholysis and nail plate separation ensue [5,17]. Splinter hemorrhages due to trauma in the older adults are most often black and found in the central or distal third of the nail plate [6]. Splinter hemorrhages may also be a sign of nail psoriasis. In a study of 220 patients >65 years, 35 subjects (16%) had splinter hemorrhages [5].

**Figure 3.**
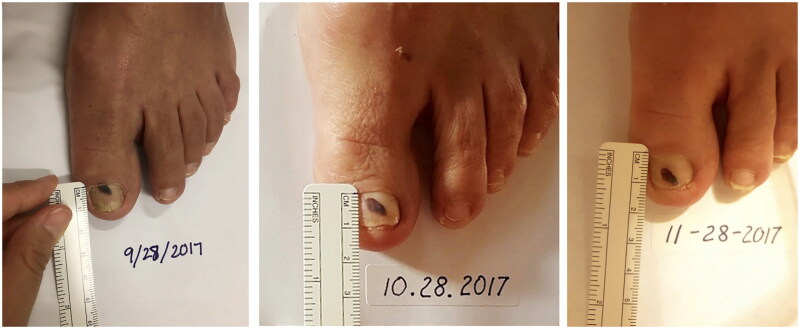
Example of a patient-initiated nail hematoma selfie of the right thumbnail on the day of examination. Patient-initiated nail hematoma selfie of the right thumbnail 1 month following the initial examination. Patient-initiated nail hematoma selfie of the right thumbnail 2 months following the initial examination 18.

The most common cause of subungual hematoma is trauma, but also may be due to foot-to-shoe incompatibility, hallux rigidus, hallux valgus, or overlapping toes [5]. In older patients, subungual hematomas/splinter hemorrhages may also be due to anticoagulant therapy [6]. A nail clipping with histopathological examination can confirm subungual hematoma. Diagnosis may also be confirmed *via* serial photography [17] (Figure 3).

Treatment includes reassurance and observation of the nail over time to ensure the hemorrhage resolves and moves distally, assuring patients that their nail discoloration is due to blood as opposed to nail melanoma [18]. In acute cases, trephination or complete removal of the nail plate to relieve pressure might help symptomatically when >50% of the nail plate is involved or >25% with fracture [19].

### Beau’s lines, onychomadesis and retronychia

**Figure 4.**
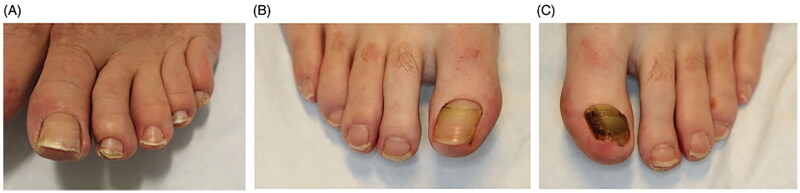
Clinical presentations of Beau’s lines, onychomadesis and retronychia. (A) Beau’s lines on the left toenails. (B) Onychomadesis of the left great toenail. (C) Retronychia of the right great toenail [20].

**Figure 5.**
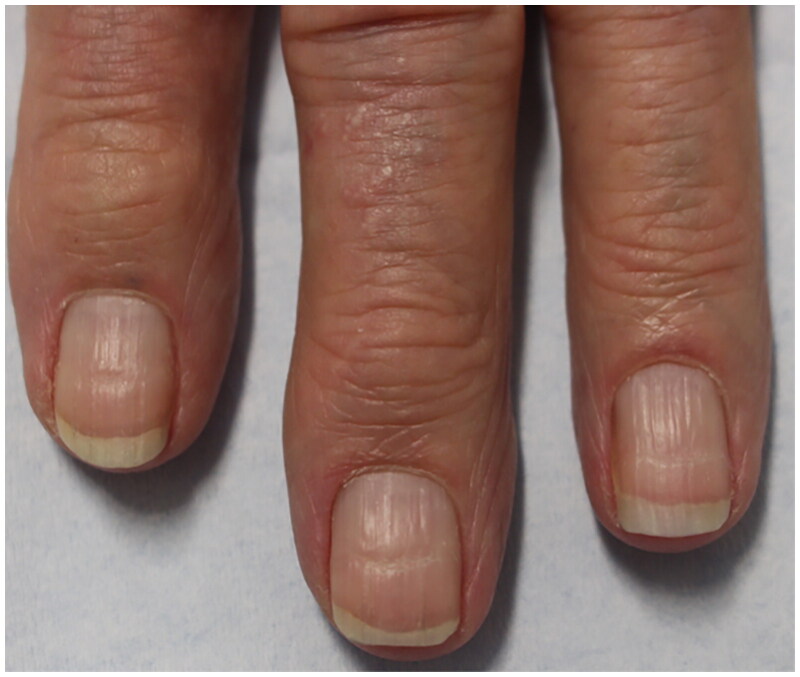
93-year-old female with bullous pemphigoid presented with Beau’s lines on all fingernails at even intervals coinciding with her monthly IVIG treatments [23].

Beau’s lines, onychomadesis, and retronychia are hypothesized to lie on a spectrum with a common pathophysiology of an insult to the nail matrix, with slowing or stopping of nail plate production (Figure 4). Beau’s lines are transverse grooves in the nail plate caused by a temporary decrease of mitotic activity of nail matrix keratinocytes [20,21]. Beau’s lines may be due to trauma, medications, or systemic illnesses [22]. When they present unilaterally, they may be caused by injury to the ipsilateral hand, wrist and elbow, nerve injury from fractures and carpal tunnel syndrome, or limb immobilization in casts, from transient decrease of blood supply to the nail matrix following trauma [20]. When Beau’s lines are due to systemic causes, such as illness, severe stress, or systemic treatment, they affect all nails [23] (Figure 5). The distance of a Beau’s line from the proximal nail fold can estimate timing of the stressor [22].

Onychomadesis is the complete nail plate separation and shedding with slow longitudinal growth rate. After a traumatic event, nail production may completely halt, leading to the loss of continuity between the nail plate and matrix. Hence, if the depression that is created from this event is deep enough, the nail will separate from the matrix and as the proximal nail grows out, it will wedge the distal plate up and eventually shed [20].

Retronychia is the malalignment of the nail plate resulting in growth of the nail plate proximally toward the nail fold. It presents as overlapping layers of nails with no longitudinal growth. It most often affects the great toenails. Retronychia may result from repeated trauma, such as running or wearing ill-fitting footwear, or a single traumatic incident. Other causes include foot static disorders, such as reflex compensatory hyperextension of the halluces [24]. There is a complete separation of the nail plate from the nail bed/matrix, with a new nail plate growing under the old one and pushing it into the nail fold, causing inflammation [20]. Complications include pain, paronychia, granulation tissue, and nail bed shortening [20].

Beau’s lines, onychomadesis, and retronychia are clinical diagnoses. Beau’s lines and onychomadesis will self-resolve once the inciting factor is removed. Patient education focuses on avoidance of trauma and keeping nails trimmed short. If retronychia is diagnosed within the first few months, patients are counseled to wear shoes with a wider toe-box to avoid toenail compression, and surgical nail avulsion may be curative. When retronychia is present for many years, treatment is challenging, and options include clobetasol ointment under occlusion to decrease inflammation and 40% urea under occlusion to chemically avulse the nail [20].

### Drug-Induced nail changes

Older patients often have medical conditions necessitating polypharmacy. Patients taking anti-inflammatory and anticoagulants such as aspirin or warfarin may develop subungual hemorrhages, affecting multiple nails in the absence of trauma [25,26]. Beta-blockers, such as propranolol, may cause digital gangrene in patients with severe peripheral vascular disease due to decreased perfusion and cardiac output due to beta-adrenergic receptor blockade [25,26]. Raynaud’s phenomenon may be the first sign of decreased perfusion, sometimes progressing to nail unit ischemia or necrosis [26].

As there is an increased prevalence of cancer and polypharmacy among older adults, patients undergoing chemotherapy treatment may experience a variety of nail changes [27]. Muehrcke’s lines are defined as opaque white transverse bands (apparent leukonychia) separated by normal pink colored nail, due to acute toxicity to tissues with high mitotic activity, such as the nail matrix. True transverse leukonychia is due to temporary impairment of distal nail matrix keratinocytes, and results in white opaque bands that are 1–2 mm wide, particularly with doxorubicin, cyclophosphamide, or vincristine. Beau’s lines, appear on all nails coinciding with timing of chemotherapy cycles. Other chemotherapy associated nail changes include longitudinal or transverse melanonychia due to activation of matrix melanocytes. Subungual hemorrhage and splinter hemorrhages may occur with taxanes and anthracyclines due to thrombocytopenia or blood extravasation. Hemorrhagic onycholysis and subungual abscesses occur in 44% of patients receiving taxanes, particularly docetaxel. Increased nail fragility and onycholysis may also occur. Paronychia with/without pyogenic granulomas are seen in association with chemotherapies including cetuximab/C225, osimertinib, and gefitinib [25,26,28].

## Infectious nail diseases

### Onychomycosis

**Figure 6.**
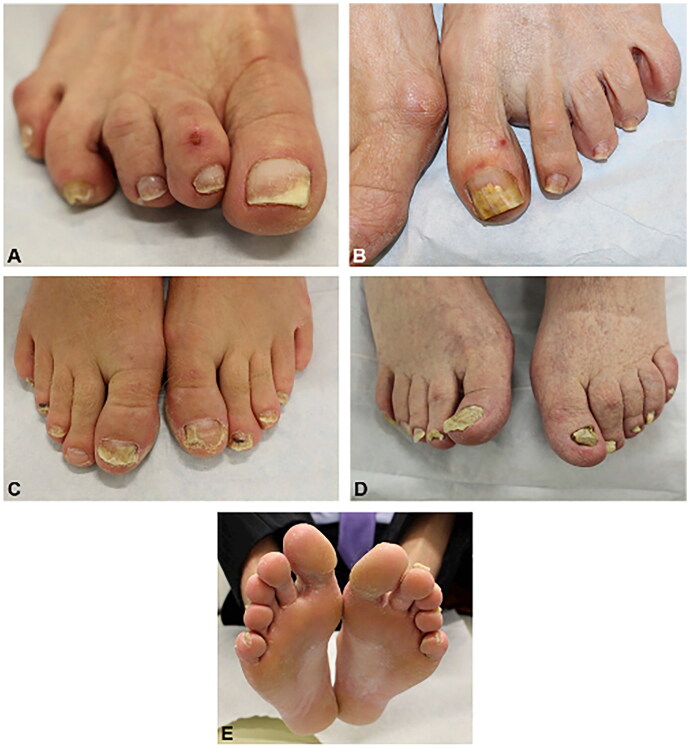
Physical examination findings in onychomycosis. A, Right great toenail with subungual hyperkeratosis and nail plate onycholysis. B, Left great toenail with yellow discoloration and onycholysis. C, Multiple toenails with subungual hyperkeratosis and onycholysis. D, Toenails with severe onychodystrophy and ridging. E, Scale on the plantar feet and web spaces [33].

**Figure 7.**
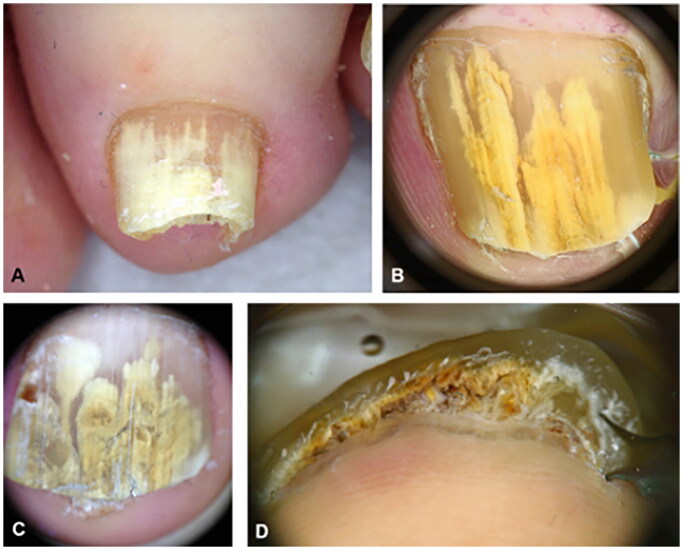
Dermoscopy of onychomycosis. A, Fringed proximal margin of the onycholysis. B, Blurred yellow-orange-brown nail discoloration in longitudinal striae (the fading mimics Aurora Borealis). C, Distribution of the discoloration in longitudinal striae or round areas. D, Ruin-like appearance of the subungual scales that are white-yellow-orange in color. Photographs courtesy of Dr Maria Bianca Piraccini [34].

Onychomycosis is a common fungal infection of the nail unit, accounting for 50% of all nail disorders [29]. It may be painful, cause psychosocial problems, lead to secondary infections and affect quality of life (QoL) [30]. Prevalence increases with age [31], and immunosuppression and diabetes mellitus are important risk factors [32]. Clinical presentation includes yellow nail plate discoloration, thickening, onycholysis, crumbling, and subungual hyperkeratosis (Figures 6,7). On dermoscopy, onychomycosis may present with a ruin-like appearance, longitudinal striae and spikes on the proximal margin of onycholytic areas, and the ‘aurora borealis’ sign which is chromonychia of multiple colors [35]. There may be scaling of the plantar feet and/or interdigital spaces (tinea pedis). A nail clipping is imperative for mycological confirmation and accurate diagnosis, especially given that many older patients present with dystrophic nails due to a variety of conditions, including nail trauma and psoriasis [29,36–38].

There are three Food and Drug Administration (FDA) approved topicals and two FDA approved systemics options for onychomycosis treatment [39]. Topicals include ciclopirox 8% lacquer, efinaconazole 10% solution and tavaborole 5% solution. FDA approved systemic agents include terbinafine and itraconazole [29,40]. Fluconazole is an off-label systemic treatment with broad-spectrum coverage [40].

Advanced age is associated with lower cure rates, likely due to slower nail growth, poor circulation, and higher frequency of non-dermatophyte mold and mixed infections compared to younger individuals [40]. Up to 20% of older patients with onychomycosis have other comorbid conditions and thus take multiple systemic medications which may interact with oral antifungals [29,41,42].

Itraconazole has many drug-drug interactions as is a potent CYP3A4 inhibitor. Terbinafine is CYP2D6 inhibitor, but has few drug-drug interactions [43]. Terbinafine is cleared both renally and hepatically, while itraconazole is cleared hepatically [40]. Topical therapy would avoid systemic side effects and drug-drug interactions, but may be less effective due to inadequate nail plate penetration [40]. Older adults may also have difficulty applying topicals if they have limited flexibility, visibility, or dexterity.

### Periungual and subungual warts

Human papillomavirus (HPV) is responsible for nail unit verruca. Immunosuppression is an important risk factor [5]. Treatment includes destructive modalities including electrocautery, cryosurgery, and ablative lasers [44], and topicals, such as salicylic acid and imiquimod [44]. Treatment of nail unit verruca is often challenging. Alternative therapies include intralesional (IL) candida antigen or bleomycin [45,46].

### Acute paronychia

Acute paronychia is defined as a bacterial infection of the nail folds and is most commonly caused by *Staphylococcus aureus.* Patients often present with erythema, tenderness, and localized pus formation. The majority of acute paronychia cases are due to trauma and typically affect one nail [3,5]. Treatment is the same in all age groups and entails incision and drainage, warm saline soaks, and systemic or topical antibiotic therapy depending on sensitivities [3].

## Environmental nail changes

### Chronic paronychia

**Figure 8.**
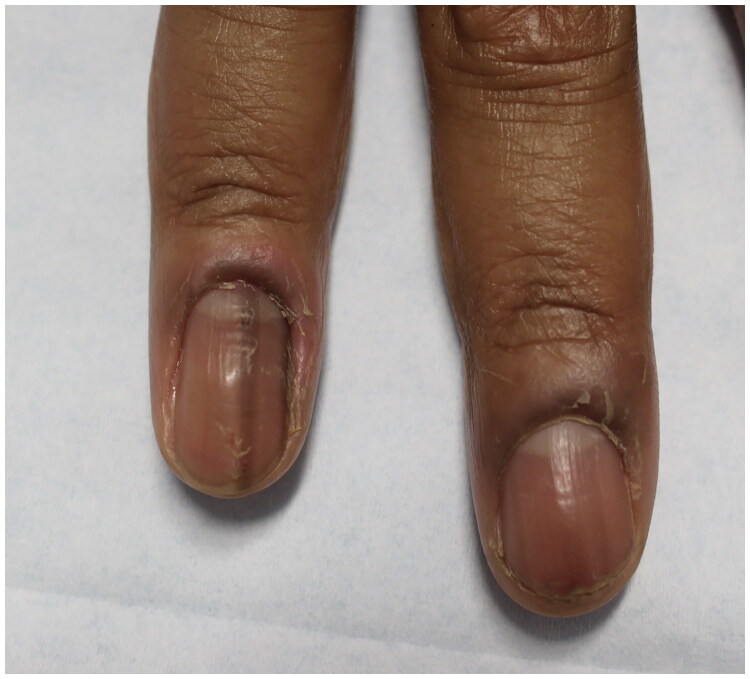
Chronic paronychia presenting with edema of the right third and fourth nail folds. Of note, there is also benign longitudinal melanonychia of the right 4th fingernail.

Older adults may develop chronic paronychia, which is caused by nail fold inflammation (Figure 8). Patients often present with erythematous and swollen nail folds with cuticle loss. In contrast to acute infections, patients may report discomfort, but are less likely to have pain [3,5]. Diagnosis is made *via* history and physical examination. Management requires irritant avoidance and keeping the digits dry. Medical treatment includes topical corticosteroids and antifungals. IL steroid treatment is sometimes used in recalcitrant cases [3,5].

### Brittle nail syndrome (BNS)

**Figure 9.**
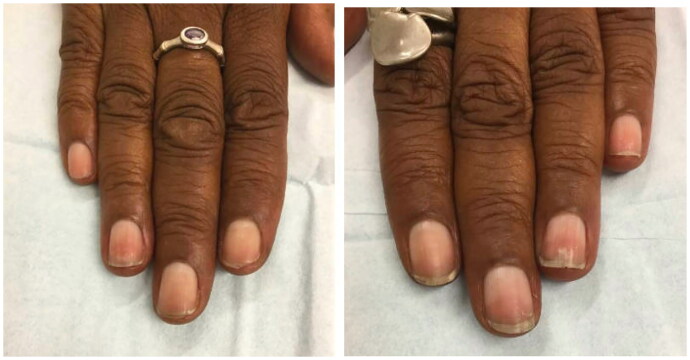
Clinical manifestations of brittle nail syndrome, including lamellar onychoschizia.

Brittle nail syndrome (BNS) is defined as increased nail plate fragility and is most frequently seen in women and older patients [47,48]. It is theorized that decreased sulfur content in results in fewer disulfide bridges in proteins forming keratin fibrils [4, 47]. The decreased cholesterol sulfate concentrations in nail clippings observed with increased age, may explain the increased incidence of BNS in older adults [4,49]. BNS presents with splitting (onychoschizia), onychorrhexis, or splitting (Figure 9). Onychoschizia is due to decreased intercellular adhesion between nail plate corneocytes, and onychorrhexis is due to impaired nail matrix function [4,6]. While BNS is often idiopathic, it is often induced or worsened by frequent handwashing, regular manicures, and trauma. Diagnosis is based on history and clinical presentation. Differential diagnoses include trauma, nail psoriasis, and onychomycosis, which can be excluded with nail clippings. An underlying cause for BNS should be ruled out, such as vitamin C or iron deficiency, hypothyroidism, and chemotherapy [50]. Treatment includes limiting contact with water and irritants, wearing gloves during wetwork, and applying nail strengtheners.

## Inflammatory nail changes

### Nail psoriasis

**Figure 10.**
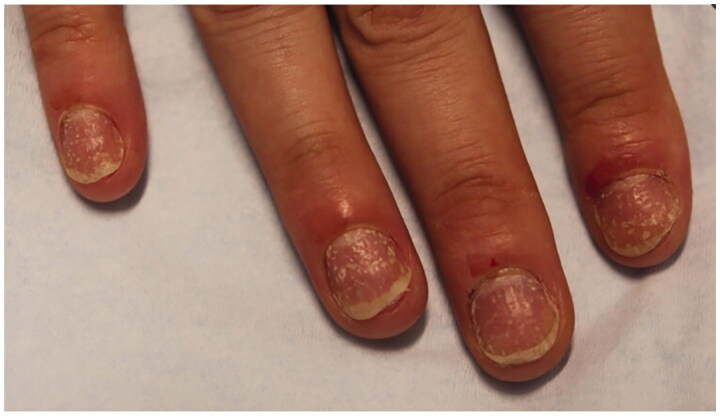
Nail pitting and onycholysis in right fingernails [20].

Nail Psoriasis (NP) is an inflammatory nail condition that may affect older patients, with a bimodal age incidence peaking at 30–39 and 60–69 years [51]. A combination of environmental, genetic and immune stressors is involved in the pathogenesis. Nail matrix psoriasis presents with pitting, crumbling, leukonychia, and red spots in the lunula, while nail bed psoriasis presents with splinter hemorrhages, onycholysis, oil drops and nail bed hyperkeratosis [52] (Figure 10). Fingernails are more frequently affected than toenails [51]. NP may present in isolation or more commonly with skin psoriasis. Nail pain is common, which can negatively impact QoL [53]. A survey-based study of 2449 psoriasis patients using the Dermatology Life Quality Index showed that there was higher impair QoL in those with vs. without nail involvement (7.2 vs. 5.3; P.001) [54]. Another survey-based study of 5400 psoriasis patients showed that NP decreased functionality most in putting on shoes or socks and household activities (21.2% and 25.1%, respectively), and caused pain in up to 35.8% of patients, further diminishing QoL [55].

Diagnosis is based on clinical history, physical examination, dermoscopy, and nail clippings. Joint examination and hand X-rays can help rule out psoriatic arthritis [56]. Treatment for NP includes topicals or IL steroid matrical injections, which may be preferable when NP is isolated to a few nails [49,57]. Systemic therapies should be considered if NP is severe, involves many fingernails/toenails and with joint involvement [57,58]. Older adults are underrepresented in NP randomized clinical trials, thus recruitment of this population in research studies are needed to establish applicable NP treatment guidelines [59].

## Neoplastic nail changes

### Nail unit melanoma

Nail unit melanoma (NUM) is a rare subset of cutaneous melanoma that is most frequently diagnosed between the ages of 50 and 70 years. The majority of NUM cases are located on either the thumb or hallux [60].

**Figure 11.**
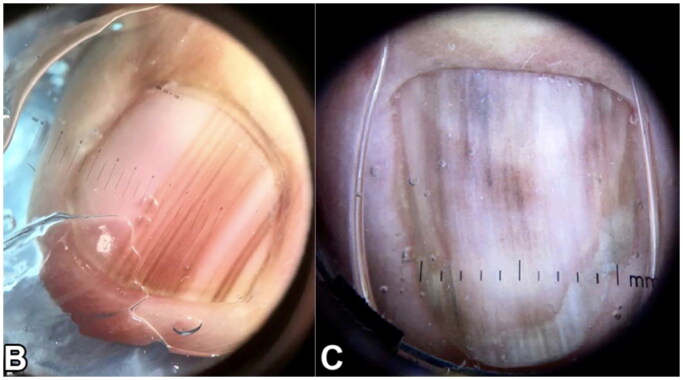
Dermoscopic appearance of subungual melanomas. B, Brown lines on a brown background, irregular color, thickness, and spacing with no loss of parallelism. C, Brown lines on a brown background, irregular color, thickness, and spacing with loss of parallelism[62].

Clinically, NUMs most commonly present as longitudinal melanonychia (LM), defined as a longitudinally oriented brown to black band that extends the length of the nail plate [61] (Figure 11). Up to 1/3 of NUMs are amelanotic, presenting as red nodules or longitudinal erythronychia with onycholysis, splitting, or ulceration [63–65]. Concerning physical examination findings are bands measuring greater than 3 mm in width, band widening or heterogeneity in color, bleeding, and nail splitting [62]. A Hutchinson’s sign, defined as periungual pigment involving the nail folds or hyponychium is often concerning for invasive melanoma [63]. Diagnosis of NUM is often delayed due to varied presentation, lack of standardized clinical approach, poorly performed biopsies, or inaccurate interpretation of histopathology [66].

NUM *in situ* can often be managed with en bloc excision that gives patients better QoL. Advanced NUM cases warrant amputation to avoid recurrence or metastases. The level of amputation at various joints is dependent on the degree of invasion into bone or joint spaces [66]. Mohs micrographic surgery may be used as a digit-sparing technique for removal of NUM, and is most commonly used for tumors measuring less than 2 mm in depth [63].

In addition to surgical management, individuals with more advanced cases of NUM may benefit from newer systemic therapies [67]. These include programmed cell death protein-1 (PD-1) inhibitor monotherapy, lymphocyte activation gene-3 inhibitor (relatlimab) or cytotoxic T-lymphocyte associated antigen 4 (CTLA-4) inhibitor (ipilimumab) [67]. Although these targeted therapies are currently the standard of care for cases of advanced cutaneous melanoma, there have been no clinical trials to date specifically analyzing response rates and outcomes in patients with NUM [68].

### Bowen’s disease

Bowen’s disease (BD), or squamous cell carcinoma *in situ*, rarely localizes to the nail unit and is most commonly diagnosed in men with peak incidence at 70 years [69]. In a retrospective review of 120 HPV-associated nail unit BD cases, HPV subtype 16 DNA was identified by polymerase chain reaction in 74% of cases [70]. Other risk factors include trauma, ionizing radiation, smoking, arsenic exposure, and chronic paronychia [6,71]. In a retrospective study of 12 cases of nail unit BD, 90% of patients were male, with mean age of onset 52 years. The thumb and middle finger were the most frequently affected digits (66%), and HPV infection was identified in 75% of cases [71].

BD most commonly presents as an ulcerated hyperkeratotic lesion often accompanied by erythema, scaling, and crusting. Pigmented forms may mimic other nail conditions, including NUM, verruca, pyogenic granuloma, subungual exostosis, glomus tumor, or lichen planus [69]. Diagnosis is often delayed due to its rarity and variable clinical presentation. Mohs micrographic surgery is the standard of care for treatment, which helps to preserve some nail unit and maintain digital function. Other nonsurgical treatment options include fluorouracil, imiquimod, photodynamic therapy, radiotherapy, carbon dioxide laser, but have lower clearance rates and higher recurrence rates compared to surgery [6,69].

## Other nail changes

### Myxoid cyst

**Figure 12.**
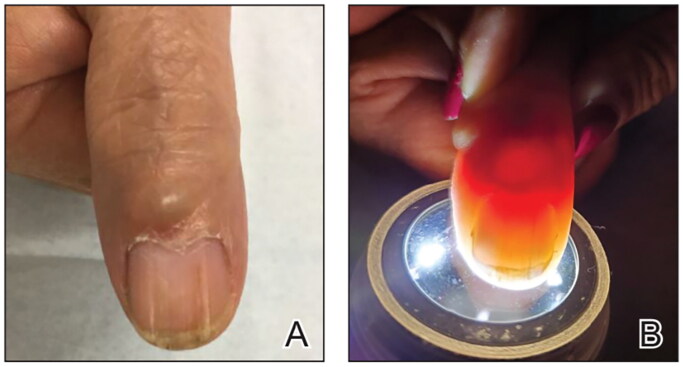
A, A translucent compressible nodule of the proximal nail fold and longitudinal groove in the nail plate of the right thumb. B, Transillumination using a dermatoscope to project light from the dorsal digit through the nail unit demonstrated a central nodule in the proximal nail fold as well as a second cyst radially. (Reprinted with permission from *Cutis*. 2020;105(2):82. ©2020, Frontline Medical Communications Inc.) [73].

Myxoid cysts affect the distal fingers and toes, and may be superficial (located near the proximal fold nail) or deep (located near the DIP joint). Clinically, they present as skin-colored to translucent, smooth, dome-shaped, and fluctuant nodules located distal to the interphalangeal joint, most commonly on the first three fingers [34,72] (Figure 12).

They most frequently affect older adults and are often associated with osteoarthritis. In a cohort study of 51 patients with digital myxoid cysts, 74.5% showed radiologic evidence of primary interphalangeal joint osteoarthritis in affected digits [34]. Due to their space occupying nature, they can influence the microvasculature, nail matrix function, nail shape and nail integrity. Consequently, when myxoid cysts are located near the proximal nail fold, they may compress the nail matrix resulting in a longitudinal groove [74]. In a retrospective case series of 34 subungual myxoid cysts, increased transverse curvature (85%), lunular discoloration (76%), and nail splitting or partial destruction (44%) were most common [74].

Asymptomatic myxoid cysts are best managed with clinical observation. Direct needle puncture with simple drainage and injection of corticosteroid may be attempted if symptomatic, although recurrence rates are high [75]. Surgical excision is an alternative.

## Conclusion

Nail conditions are common in geriatric patients and may impact their daily lives. Managing these conditions may be even more challenging in older adults due to limitations in their mobility dexterity when applying treatments. Moreover, even with proper diagnosis and treatment, resolution is often slow, since nails grow even more slowly in older adults. Examination of the toenails should be performed during the overall foot exam of older patients, especially considering that many comrobidities that affect older adults, such as diabetes, peripheral neuropathy, and peripheral artery disease, commonly involve the feet [76]. As nail changes can physically and psychologically affect patients, it is important for physicians to diagnose and manage these nail conditions that can so easily go unrecognized in this expanding patient population.

## Data Availability

Data sharing is not applicable to this article as no new data were created or analyzed in this study
